# Patent Medicine Dealers and Irrational Use of Medicines in Children: The Economic Cost and Implications for Reducing Childhood Mortality in Southeast Nigeria

**DOI:** 10.1371/journal.pone.0091667

**Published:** 2014-03-12

**Authors:** Benjamin S. C. Uzochukwu, Obinna E. Onwujekwe, Chinenye Okwuosa, Ogochukwu P. Ibe

**Affiliations:** 1 Department of Community Medicine, College of Medicine, University of Nigeria Enugu-Campus, Enugu, Nigeria; 2 Department of Health Administration and Management, College of Medicine, University of Nigeria Enugu-Campus, Enugu, Nigeria; 3 Health Policy Research Group, College of Medicine, University of Nigeria Enugu-Campus, Enugu, Nigeria; Groningen Research Institute of Pharmacy, Netherlands

## Abstract

This study assessed the economic costs of irrational medicine use by Patent Medicine Dealers (PMDs) for malaria, acute respiratory infection (ARI) and diarrhea diseases (DD) in Nigeria. Exit interviews were conducted with 395 respondents who sought care for their children from 15 PMDs in Abakpa district of Enugu state Nigeria. Of the total respondents, 80.0% received treatment for malaria while 12.0% and 8.0% received treatment for DD and ARI respectively. The average number of drugs dispensed per patient was 6.8, average percentage of patients given injections was 72.5%, average percentage of patients given one or more antibiotics was 59.7%, while the percentage of patients given non essential drugs was 45.9%. The additional costs to the standard treatment in Naira was 255, 350 and 175 for malaria, ARI and DD respectively. The losses attributable to irrational dispensing was 4,500 Naira. However, more than half of the drugs were on essential drug list, implying some cost savings for the consumers, but the high number of drugs (6.8) on average/patient is likely to increase the total cost of drugs cancelling out the cost savings to consumers arising from dispensing essential drugs.

## Introduction

Diarrhea diseases (DD), acute respiratory infection (ARI) and malaria cause more than half of childhood mortality in some developing countries and irrational prescribing of drugs has been noted as a major health concern in these countries. [1], [2]. In Nigeria, many children under the age of five die from these diseases that are preventable or treatable with low cost drugs and these drugs are usually bought from patent medicine dealers (PMD) that have constituted the primary source of drugs in rural and urban Nigeria especially for the poor [3]–[5].

The patent medicine dealer has been defined as “a person without formal training in pharmacy and sells orthodox pharmaceutical products on a retail basis for profit” [6]. They dispense drugs most of the time but do not prescribe. Geographical accessibility, free consultation, lower cost, shorter waiting times, drug availability, longer opening hours, friendly staff disposition, greater confidentiality, and flexible payment modes have been adduced as some of the reasons for preferring PMDs [2], [7]. However, it has been noted that these PMDs have poor knowledge of childhood illnesses and dispensing behaviour especially with malaria episodes [8].

Rational use of medicines requires that “patients receive medications appropriate to their clinical needs, in doses that meet their own individual requirements, for an adequate period of time, and at the lowest cost to them and their community” [9]. Irrational or non-rational use is therefore the use of medicines in a way that is not compliant with rational use as defined above. Aspects of irrational medicine use are found in diagnosis which includes (inadequate examination of patient, incomplete communication between patient and doctor, lack of documented medical history, inadequate laboratory resources); prescribing which includes extravagant prescribing, over-prescribing, incorrect prescribing, under-prescribing and multiple prescribing); dispensing which includes (incorrect interpretation of the prescription, retrieval of wrong ingredients, inaccurate counting, compounding, or pouring, inadequate labelling, unsanitary procedures, packaging, poor-quality packaging materials, odd package size, which may require repackaging and unappealing package) and patient adherence which includes (poor labelling, inadequate verbal instructions, inadequate counselling to encourage adherence, inadequate follow-up/support of patients, treatments or instructions that do not consider the patient’s beliefs, environment, or culture [10].

Evidence from Kazakhstan shows that there is increasing trend in use of expensive, widely advertised, brand name medications, and reduction in the use of generic first line medications thus leading to adverse health and economic effects [11]. The effects of irrational use of medicines include fast development of resistance, treatment failure, high incidence of toxicities, poor health outcomes and increased health care costs [10]. A study in Nepal of primary health care centers showed that 20%–52% of drug costs were wasted through irrational prescribing [12]. The wastage was incurred by formally trained health workers and it is expected to be worse with PMDs who have no formal training on treatment of common ailments. Studies have shown that these practices could be improved through a combination of different approaches such as training and capacity building [13], [14].

Studies of irrational medicine use have focused on formal healthcare facilities and with clinicians or formal health care workers [15]–[17], but little has been done in the informal health care facilities. And over the years little or no attention has been focused on the cost of irrational medicine use especially in the informal healthcare facilities.

There is paucity of information in Nigeria on the economic costs associated with inadequate medicine dispensing for childhood illnesses by PMDs. Information on this is therefore needed especially against the background that irrational use could have great impact on the household income where a large proportion of a family’s out-of-pocket expenditures on health care is on drugs [18]. This study therefore assessed the economic costs of irrational medicine dispensing by patent medicine dealers for malaria, ARI and DD where most of Nigerians seek for care as a first resort. The result is expected to inform policy makers on the need for interventions to improve rational use of medicines in both formal and informal health facilities.

## Materials and Methods

### Study Area

This study took place in Abakpa district of Enugu east local government area (LGA) of Enugu state South east Nigeria. Abakpa is the most densely populated of the 4 districts that make up Enugu east LGA with a projected population of 137,726 [19]. It has both public and private health care providers including a primary health care centre with well established childhood immunization services. There are 45 patent medicine dealers, several private hospitals, pharmacies and laboratories. Other sources of treatment are traditional healers and public hospitals outside the district. The native population is predominantly Igbo with a sprinkling of Hausa and Yoruba communities. The inhabitants are mainly civil servants and traders with Christianity as their main religion.

### Study Design

This was a cross sectional descriptive study conducted to ascertain the cost of irrational medicine dispensing in children with malaria ARI and DD using patent medicine dealers in an urban community. A census of all the patent medicine dealers was conducted by 2 community members and 2 researchers with the assistance of the patent medicine dealer’s association chairman. The chairman was brought in to gain support and cooperation of the patent medicine dealers and 45 PMDs were identified.

### Sampling and Sample Size

Using simple random sampling technique, 15 PMD facilities were selected from a sample frame of the 45 PMD facilities. And from these we collected information from between 25 to 30 exiting mothers. Thus, a total of 395 mothers/caregivers who exited the facilities and did not have a prescription from a formal health worker were intercepted and the medicine dispensed to their sick children were collected, observed and recorded. They were also asked if their children were given any injections. Only fresh cases were interviewed once each day at these facilities and no re-attendances were taken or interviewed twice.

### Data Collection

Pretested interviewer-administered questionnaires were used to elicit information from 395 respondents who sought care for their children as they exited from the 15 PMD outlets and after obtaining verbal consent. The information sought were respondents’ and patients’ socio-demographic characteristic, number and type of medicines dispensed and for which disease and whether they had a prescription or not from a health worker. Fever was used as a proxy for malaria; cough and difficulty in breathing for ARI and watery stool for DD. For each of the treatment provided, the variables collected were the average number of medicines dispensed per encounter, percentage of patients with injections, average percentage of patients with one or more antibiotics dispensed, percentage of patients with essential drugs and average cost of drugs per patient for the medicines dispensed.

A total of 50 patients who had irrational medicine use in form of dispensing were randomly selected and followed up after 2 weeks to determine further costs incurred as a result of the illness (consultation fees, drugs, transport and the cost equivalent to the working days lost due to illness). Market price was used to cost the drugs.

### Data Analysis

Each treatment episode without a prescription from a formal health worker was analyzed based on the symptoms reported by the caregiver and the treatment provided at each episode. These episodes were assessed by the researchers who were all medical doctors from the University of Nigeria teaching hospital Enugu. The Nigerian standard treatment guideline for Malaria, ARI and DD were used to determine the adequacy of treatment [20] and any practice outside this was regarded as irrational.

The data analysis was done at two levels. The first analysis compared average number of medicine items received per patient, average percentage of patients who received an injection from the PMD facilities, average percentage of patients who received an antibiotic, percentage of patients who were dispensed non-essential drugs and average number of medicines per patient. The second analysis compared the additional costs to the standard treatment for the different ailments. Costs of drugs dispensed to the caregiver were determined using the prices obtained from the market. Cost of needles and syringes and cotton swabs were incorporated for those who received an injection. Total cost of dispensed drugs and average drug cost per patient were calculated. The difference between the average cost of the standard treatment and the medicines purchased was regarded as the additional cost. The additional direct and indirect costs to the standard treatment for the different ailments for the 50 patients that were followed up was calculated by computing the further costs incurred as a result of the illness (consultation fees, drugs, transport and the cost equivalent to the working days lost due to illness). Both analyses were run with EPI-Info and Excel.

### Ethical Approval

Approval was sought and received from the ethics committee of the College of Medicine University of Nigeria Enugu-campus. Verbal consent was sought from all the respondents. The ethics committee approved of the consent procedure. Verbal consent was used before information was collected from the respondents because previous experiences have shown that respondents are reluctant to sign documents. Reasons are that it might be used for tax assessment purposes or the information might affect their businesses. To ensure that verbal consent was sought, the field workers had a register for household numbers and participants who gave consent were ticked. The authors also verified that this process was observed in the field.

## Results

### Socio Demographic Variables of Respondents

As shown in table 1, out of 395 respondents, 365 (92.4%) were females. Among the ill persons, 249 (63%) were females. The mean age of the respondents was 34.1 years ±6.5 SD and for the ill persons, it was 3.8 years ±1.7SD. Also, of the total respondents, 316 (80.0%) received treatment for malaria, 47 (12.0%) received treatment for DD and 32 (8.0%) received treatment for ARI.

**Table 1:** Socio demographic variables of respondents.

Variables	N (%)N = 395
Sex of respondent (Female)	365 (92.4)
Years of education of respondent: Mean	9.5 (3.6)
Age of respondent (Years): Mean (SD)	34.1 (6.5)
Age of ill person (Years) Mean (SD)	3.8 (1.7)
Sex of ill person (Female)	249 (63.0)
% Received treatment for malaria	316 (80.0)
%Received treatment for DD	47 (12.0)
% Received treatment for ARI	32 (8.0)

### Drug Dispensing Pattern as Practiced by the Patent Medicine Dealers

Table 2 shows that the average number of drugs received per patient was 6.8, average percentage of patients with injections was 72.5%, average percentage of patients with one or more antibiotics was 59.7% while percentage of patients with non essential drugs was 45.9%.

**Table 2:** Drug dispensing pattern as practiced by the patent medicine dealers.

Drug use indicators	
The average number of drugs per patient	6.8
Average percentage of patient with injection	72.5%
Average percentage of patients with one or more antibiotics	59.7%
Percentage of patients with non essential drugs	54.9%

### Additional Costs to the Standard Treatment for the Different Ailments

As shown in table 3, the additional mean costs to the standard treatment in Naira were as follows: malaria 255 (164.5 SD); ARI 350 (201.6 SD) and DD 175 (93.5 SD). The losses attributable to irrational dispensing/patient in Naira was 4,500 (1950 SD) with the direct costs being 1950 Naira and indirect costs being 2550 Naira. Drug costs constituted 90% of the direct costs.

**Table 3:** Additional costs to the standard treatment for the different ailments (Naira).

Variables	Mean (SD)
Malaria	255 (164.5)
ARI	350 (201.6)
DD	175 (93.5)
Additional cost attributable to irrational dispensing/patient for 50 patients followed up	
Direct costs	1950 (924.6)
Indirect costs	2250 (1108.2)
Total costs	4,500 (1,950)

The mean values are in Naira, 150 Naira = I US Dollars.

## Discussion

In this study, patients incurred additional costs as a result of irrational dispensing of medicines and this is more with patients presenting with ARI who incurred the highest additional costs. This is worrisome, considering that losses attributable to irrational dispensing per patient is on the average catastrophic as more than two thirds (64.4%) of the Nigerian population live below the poverty line of US$1 per day [21], [22]. Rational medicine use entails not only that appropriate drugs are given, but that they are available when needed and at affordable price [23]. However, differences have been noted to exist in drug use which could be due to differences in skills on the part of prescribers, differences in ownership of health facilities and lack of access to correct information on proper use of medicines [24], [25]. This underscores the need for professional associations like the association of patent medicine dealers to take leadership role in promoting proper use of medicines to their members.

From the study, a small number of patients had a prescription which shows that majority rely on self or presumptive treatment. Also the average number of drugs per patient of 6.8 is higher compared to International Standards of 1.4–2.0 drugs per prescription [24]. Thus, the major problem associated with drug use in these facilities is over-dispensing. In several developing countries, medicines are not always available in public hospitals and primary health centres. This has led to PMDs becoming the primary source of orthodox drugs in both rural and urban communities in Nigeria [25], [26]. although this does not mean that even if they were available that PMDs would not still be the primary source. Poor perceptions of public services provision may be more important reasons. In Nigeria, studies have shown that the minimum educational attainment of these PMDs is primary schooling [27]–[30] and the pharmacy laws in the country does not specify the educational level to be attained before the patent medicine dealers’ license is issued [31]. And this minimal level of education has been found not to confer on the PMDs or apprentice, correct knowledge about the drugs at their disposal or the common illnesses experienced by customers [32]–[35]. Patent medicine dealers have also been noted to have poor knowledge and poor dispensing behavior in relation to childhood disease episodes [6] and evidence exists in Nigeria that health personnel tend to embark on poly pharmacy in their attempts to treat a number of diseases simultaneously [36].

This study shows that the average percentage of patients with injections was high. Patent medicines license does not allow PMDs to stock injectables, let alone dispense them, yet injections are noted as constantly being overused in Nigeria [36]. The transmission of HIV through needles sticks or injections contaminated with material from an infected individual is well established [37]. Also, the percentage of patients with one or more antibiotics was high in this study, although this may have been for the ARI. It has been noted that the most commonly prescribed and dispensed drugs for children are antibiotics when in most cases these children suffer from viral infections [38].

The only drugs the PMDs are authorized to sell are over-the counter (OTC) drugs. These OTC drugs can be purchased without a prescription and are commonly used to treat symptoms of common illnesses that may not require the direct supervision of a physician such as analgesics (paracetamol and panadol), multivitamins etc [39]. However, in practice they sell all types of drugs depending on their financial capability to procure them [40]. Also the primary function of the PMD is a business and like any other business person, the PMD tries to respond to customer demand. Thus the bulk of customer-PMD interactions in some developing countries have been noted to follow patient’s demands and expectations as well as profit motive [6], [41]. And as have been noted elsewhere, this definitely leads to a waste of scarce resources on the part of the patient which could have been deployed to other uses [42].

A positive finding is that more than half of the drugs were on essential drug list, implying some cost savings for the consumers. But the high 6.8 drugs on average/patient are likely to increase the total cost of drugs cancelling out the cost savings to consumers arising from dispensing essential drugs.

A limitation of this study is that we could not present the prescribing indicators by prescription and OTC and for the three tracer diseases since our main concern was drug dispensing. These are possible areas for future studies.

In conclusion, the study provides evidence that the average number of drugs, antibiotics and injections dispensed per patient by PMDs in Nigeria is unacceptably high. This imposes unnecessary cost (often out-of-pocket payments) on health care users especially the poor who are more prone to diseases/ill health. Thus expenditure that could have been used by caregivers for other healthcare benefits is being wasted under irrational dispensing. The study also presents useful data for policy makers on costs of irrational medicine use which is something seldom done.

Therefore, an intervention area by policy makers is the training and re-training of the PMDs on rational medicine use especially dispensing practices. There is also need to strengthen the PMD’s regulatory system by government. Also, consumer information on appropriate drug consumption through community health education is likely to reduce the cost of treatment to caregivers. Ensuring that medicines from the essential drug list are used especially at the primary health care level is likely to save cost both to the individual and the health systems. This is imperative in order to achieve the MDG goal of reducing infant mortality in Nigeria by the year 2015.

### Limitation of the Study

In this study, the economic cost is limited only to losses attributable to irrational dispensing. We did not take into account the cost incured from the effect of irrational dispensing which include: development of resistance, treatment failure, high incidence of toxicity, risk of drug interaction and which will result into poor health outcome and eventual economic cost. We acknowledge this as a limitation of the study and a potential area for future studies.
